# Reporting on the Acceptance and Usability of a Virtual Reality Visual Rehabilitation Program in Pediatric Patients With Homonymous Hemianopia

**DOI:** 10.1089/jmxr.2024.0007

**Published:** 2024-07-17

**Authors:** Danielle Tchao, Eduardo Garcia-Giler, Kyle Cheung, Bill Zhang, Inci Yaman Bajin, Mariana Misawa, Monica Daibert-Nido, Eric Bouffet, Michael Reber, Lora Appel

**Affiliations:** 1 OpenLab, University Health Network, Toronto, Canada.; 2 Department of Cell and System Biology, University of Toronto, Toronto, Canada.; 3 Donald K. Johnson Eye Institute, Krembil Research Institute, University Health Network, Toronto, Canada.; 4 Laboratory Medicine Pathobiology, University of Toronto, Toronto, Canada.; 5 Division of Hematology/Oncology, The Hospital for Sick Children, Toronto, Canada.; 6 Department of Ophthalmology and Vision Sciences, University of Toronto, Toronto, Canada.; 7 Faculty of Health, School of Health Policy and Management, York University, Michael Garron Hospital, Toronto, Canada.

**Keywords:** virtual reality, hemianopia or hemianopsia, rehabilitation, low-vision, personalized medicine

## Abstract

**Background/Objectives:**

Visual field defects are common in patients with brain tumors. In children, early vision loss can lead to delayed development of motor, language, and social skills, limiting their independence and increasing the risk of depression or anxiety later in life. There is emerging evidence that audio–visual stimulation in the blind field is an effective rehabilitation technique for treating visual field loss. The purpose of this article is to report on feedback from participants in a pilot study that explored virtual reality (VR) as a home-based visual rehabilitation tool for treating hemianopia patients.

**Subjects/Methods:**

Ten pediatric patients with homonymous hemianopia (ages 10–17 years) were recruited for the study conducted through Toronto Western Hospital. Participants followed an audio–visual stimulation protocol from home over 4–6 weeks, training their visual perception through an head-mounted display Meta Quest 2 VR headset for 15 min, every other day. Participants completed visual assessments at baseline, postintervention, and at 1- and 6-month follow-ups. A semi-structured interview was conducted postintervention to collect feedback on the participant’s experience with the device and the training protocol.

**Results:**

This article reports on the data collected through the exit interviews. All participants were able to complete the visual training protocol, and reported few technical difficulties using the system as well as few symptoms of cybersickness. Half of the participants perceived an improvement in their visual perception after completing the intervention. Participants were generally self-motivated to adhere to the therapy, but a common complaint was that the training became repetitive and they lost interest as the weeks progressed. Personalization or gamification of the intervention was recommended by participants for improving engagement.

## Introduction

Cancer is the leading cause of disease-related death among children aged 0–14 years in the United States,^1^ with brain tumors replacing leukemia by 2014 as the leading type of cancer causing death among the pediatric population.^2^ As survival rates improve for patients with brain cancers owing to better detection and treatment techniques,^3^ more focus is shifting toward management of long-term complications arising from the disease or treatment itself. In a recent study from the Children Cancer Survivor Study, 22.5% of survivors of childhood astroglial tumors had visual impairment.^4^ Visual impairment includes decreased visual acuity and contrast sensitivity, color vision loss, and visual field loss, which are often underdiagnosed. Seventy nine percent of individuals with brain tumors who have visual field defects present hemianopia (homonymous, bitemporal, or quadrantanopia), which is a loss of conscious vision owing to damages along the optic tract.^5,6^ Hemianopia can lead to delayed motor and language skills, or impede social and cognitive development for children which can greatly reduce their quality of life (QoL).^7,8^ Vision loss can have a significant impact on a person’s ability to read, recognize faces, or manipulate small objects, limiting their independence in carrying out instrumental activities of daily living (e.g., driving, preparing meals, using a smart phone)^5,7^ reducing their opportunities for employment and social engagement, altogether increasing their risk for depression and anxiety.^9^

There is emerging evidence that audio–visual stimulation in the blind field is an effective rehabilitation technique for treating visual field loss, such as hemianopia.^10–14^ Traditional audio–visual stimulation requires the use of specialized equipment in a clinical setting, but advanced technologies such as virtual reality (VR) are being investigated to provide more effective and accessible solutions to patients. Health care is increasingly taking advantage of VR, and specifically with pediatric patients given their relative familiarity/acceptance of the technology and the ability of this technology to employ gamification. In particular, VR is being used as a distraction tool in the management of pain and anxiety for burn wound care, dental treatment, vaccine/injections, and during preoperative procedures.^15–18^ Our technology, Re:Vision, is delivered as a home-based, remotely controlled, audiovisual stimulation procedure. Participants are not required to commute/travel for clinical appointments, decreasing the number of visits to the clinic and therefore the burden of disease.

VR holds great therapeutic potential in vision care because of its ability to simultaneously stimulate visual and auditory senses. However, adoption rates of VR into clinical practice remain low owing to implementation challenges,^19,20^ as well as personal perceptions and attitudes of health care providers toward the technology.^21^ Still, recent Food and Drug Administration approvals of two new digital therapeutics for amblyopia^22,23^ support the use of VR in visual rehabilitation. Such portable devices also facilitate rehabilitation from the patient’s home, increasing adherence to protocols that in turn improves clinical outcomes. Alongside clinical effectiveness of vision therapeutics, it becomes essential to understand the usability challenges of VR technology and the feasibility of administering this type of protocol in living spaces.

### Objectives

This work is an additional analysis of the preprinted study of Misawa et al.^24^ The primary objective of this analysis was to identify challenges and successes of a VR-based visual rehabilitation intervention when administered in participants’ private residences and to determine key areas for improving the VR protocol for our follow-up trial.

A secondary objective was to determine the perceived (self-reported) impact on visual perception to better design outcome metrics that also capture meaningful change in participant’s every-day-life for future larger studies.

## Methods

A study led by investigators from The Hospital for Sick Children and the Krembil Research Institute was conducted between July 2022 and October 2023 to evaluate the impact of using a VR-based therapeutic intervention (Re:Vision) to improve visual perception in the blind field for hemianopia patients. The study protocol was approved by the Research Ethics Boards at The Hospital for Sick Children in Toronto, Canada (Sickkids REB# 1000076413) and at University Health Network, Toronto, Canada (UHN REB# 21–5978), and registered on clinicaltrials.gov (NCT05065268). Written informed consent was obtained from all participants in the study.

Participants in the trial used the Meta Quest head-mounted display (HMD) from home, every other day for a 4–6-week period, to perform audio–visual stimulation involving 3D multiple object tracking. Clinical outcomes of the study are reported in a separate complementary article.^24^ At the end of study, we conducted exit interviews with participants and their parents to collect feedback about the technology, training, and protocol. In this article, we report only on the data obtained from the exit interviews.

### Participants

All participants (*n* = 10) were pediatric patients from the Hospital for Sick Children diagnosed with homonymous hemianopia.^24^ Clinicians from the same hospital recruited patients with or without lesions involving the optic chiasm, with or without active chemo-treatment, and for time of diagnosis ranging from 24 to 192 months. Participant demographics are described in Table 1.

#### Inclusion criteria

1. Homonymous hemianopsia
2. Male and female
3. 8–18 years of age
4. Interpupillary distance ≥56 mm
5. Best corrected visual acuity ≥20/20
6. Ability to follow the visual and auditory stimuli and training instructions
7. Home Wi-Fi access

#### Exclusion criteria

1. Ocular diseases
2. Both eyes with media opacity that impairs microperimetry testing
3. Inability to perform during testing and training
4. Consumption of psychoactive drugs
5. Three consecutive scores <25 at inclusion using the Virtual Reality-Induced Symptoms and Effects (VRISE) scale
6. History of vertigo or dizziness
7. Prior vision rehabilitation interventions

### Intervention and assessments

Re:Vision consisted of training sessions performed every second day from home using a Meta Quest 2 HMD over a period of 4–6 weeks. Each training session was made up of three blocks of stimulation; each block lasted approximately 5 min. The stimulation involved 3D multiple-object tracking where, in the virtual environment, participants were asked to track a moving ball (temporarily highlighted) among other similar balls designed to act as distractors. After 20 s of motion, participants were asked to select the originally highlighted ball by using the VR hand controller. This activity was repeated throughout the stimulation, with the training software automatically increasing or decreasing the speed of the balls based on whether the participant’s previous selection was correct or incorrect (dynamic 1:1 staircase procedure).^25^ In the first 2 weeks of stimulation (“flexible” training), participants were allowed to move their eyes and/or head to help with tracking the moving targets. Subsequently, a central fixation point was introduced; for the remainder of the intervention period (“fixation” training), participants were asked to fixate their eyes on the point and track the moving objects using only their peripheral vision.

**FIG. 1.**
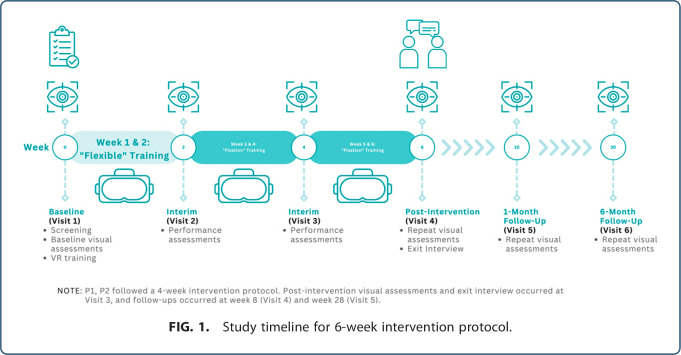
Study timeline for 6-week intervention protocol.

Visual assessments were conducted by an ophthalmologist at Toronto Western Hospital at baseline, at the end of the intervention period, at the 1-month follow-up, and at the 6-month follow-up (Fig. 1). The duration of each in-clinic assessment was approximately 1.5 h.

### Virtual Reality-induced symptoms and effects

At baseline (Visit 1), all participants were screened for possible symptoms of cybersickness (VRISE questionnaire) resulting from the use of VR.^26^ This short questionnaire is made up of five questions, each scored from 1–7, and was completed within the HMD by using the hand controller. A lower score corresponds to a higher degree of symptoms and only participants who scored ≥25 were included in the study.^24^ During the intervention period, participants were automatically prompted to complete the VRISE questionnaire immediately before and after each at-home training session. Any participant who indicated a posttraining score of <25 on three consecutive sessions would be withdrawn from the study.

### Semi-Structured exit interview

All participants (*n* = 10) took part in an in-clinic semi-structured exit interview with a research coordinator after completing their intervention. The interview took an average of 18 min and was composed of 18 questions, including both open-ended questions and quantitative ratings covering topics such as *technical training*, *system setup*, *system usability*, *device comfort*, *acceptance of the protocol*, and *overall experience*. See Appendix A for the complete interview script. Although questions were directed at the participants, a parent was present during the interviews (required as participants were minors) and sometimes provided additional feedback or clarification after their child responded.

### Analysis

All exit interviews (*n* = 10 participants) were audio-recorded with consent and transcribed by the research coordinator. Transcriptions were compared with hand-written notes taken in real time during the interviews and the data was consolidated into an Excel spreadsheet for analysis. Data were manually coded and compared to supporting context from prior email correspondence with participants where applicable (e.g., troubleshooting technical issues, rescheduling appointments to accommodate summer vacations/summer camp). All open-ended responses underwent thematic analysis and quantitative ratings were analyzed using descriptive statistics.

## Results

**FIG. 2.**
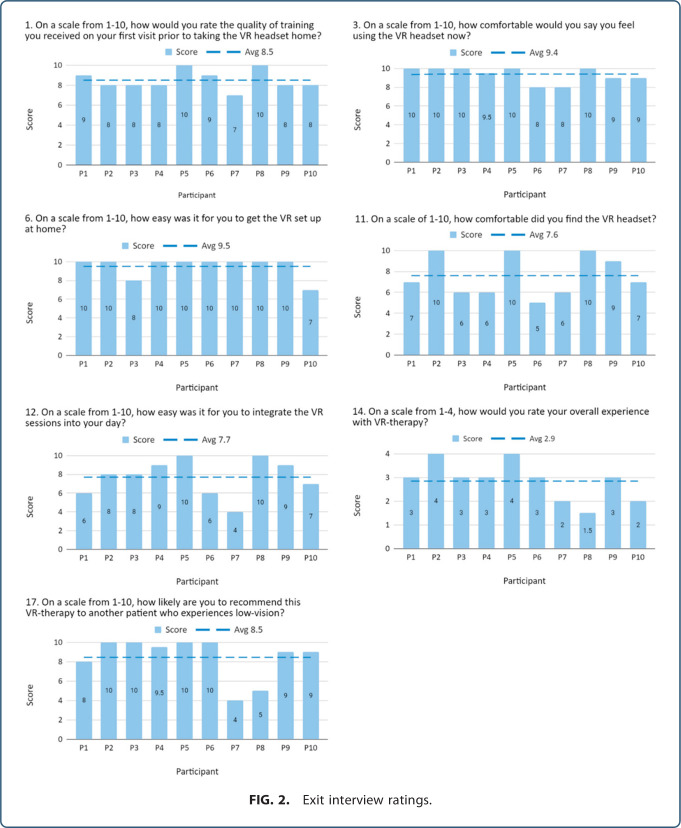
Exit interview ratings.

Results for all quantitative ratings from the exit interviews are presented in Table 2 (Exit Interview Ratings-Means and Mode) and Figure 2 (Exit Interview Ratings). Qualitative feedback addressing technical training, system setup, system usability, device comfort, adherence to the protocol, and overall experience is summarized in the sections that follow.

**Table 1.** Participant Demographics

Participant	Sex at Birth	Age (years)	Time from Diagnosis (years)	Visual field deficit	Location of Tumor	Active chemo/targeted Treatment
P1	F	16	9	Right HH^a^ + BTH^b^	Chiasmatic	Yes
P2	M	17	10	Right HH^a^	Chiasmatic	Yes
P3	F	15	13	Right HH^a^	Posterior	Yes
P4	M	15	6	Left HH^a^	Posterior	No
P5	M	16	17	Left HH^a^	Chiasmatic	No
P6	M	13	4	Right HH^a^	Posterior	No
P7	M	13	6	Right HH^a^	Chiasmatic	Yes
P8	M	10	8	Right HH^a^	Posterior	No
P9	M	16	2	Left HH^a^	Chiasmatic	No
P10	F	11	6	Left HH^a^	Posterior	No

a Homonymous hemianopia.

b Bitemporal hemianopia.

**Table 2.** Exit Interview Ratings—Means and Modes

Question	Mean^a^	Mode
**Q1. On a scale from 1 to 10, how would you rate the quality of training you received on your first visit before taking the VR headset home?** 1 = Training Was Poor/Insufficient; 10 = Training Was Thorough/Complete	8.5	8
**Q3. On a scale from 1 to 10, how comfortable would you say you feel using the VR headset now?** 1 = Not Comfortable at All; 10 = Very Comfortable	9.4	10
**Q6. On a scale from 1 to 10, how easy was it for you to get the VR setup at home?** 1 = Very Challenging; 10 = Very Easy/Straightforward	9.5	10
**Q11. On a scale of 1 to 10, how comfortable did you find the VR headset?** 1 = Not at All Comfortable; 10 = Very Comfortable	7.6	6, 10
**Q12. On a scale from 1 to 10, how easy was it for you to integrate the VR sessions into your day?** 1 = Very Challenging; 10 = Very Easy	7.7	6, 8, 9, 10
**Q14. On a scale from 1 to 4, how would you rate your overall experience with VR therapy?** 1 = Did Not Want to Continue Therapy (Stop Early) 2 = Needed Encouragement to Keep Up With Therapy 3 = Mostly Self-Motivated to Keep Up With Therapy 4 = Looked Forward to Therapy Each Session	2.9	3
**Q17. On a scale from 1 to 10, how likely are you to recommend this VR therapy to another patient who experiences low vision?** 1 = Definitely Not Recommend; 10 = Highly Recommend	8.5	10

a All ratings were on a scale from 1 to 10 except for Q14 which was on a scale from 1 to 4.

### Cybersickness

Broad references to VRISE were made as part of the exit interview. The complete quantitative data from VRISE questionnaires are described in detail in our complimentary article^24^ that reports on the clinical outcomes of this trial. Safety assessed using these scores indicated no adverse events related to using the intervention with only one postintervention score <25 and average scores of 33.1 ± 2.8 out of 35 postintervention.^24^

### Technical training

At the first clinic visit, each participant was given technical training by a member of the research team on how to configure and operate the VR device and used the HMD to practice a demo version of Re:Vision before taking the equipment home. A handout with simple How-To instructions and contact information for the lab manager was sent home along with the equipment. Participants reported that the training and materials they received were “*straightforward*” (P2, P5) and “*simple*” (P7), and that there was no need for extra handouts or a study website as part of the training. In three cases, participants mentioned they had expected a different number of stimulation blocks during their at-home intervention sessions than what they experienced (two versus three blocks); one case was owing to a required software update which was addressed by the research lab. Nonetheless, the quality of the technical training provided at the clinic was rated high overall by participants (Q1 mean = 8.5, M_o_ = 8, scored out of 10).

Participants reported that they felt very comfortable operating the Meta Quest 2 by the end of the intervention period (Q3 mean = 9.4, M_o_ = 10, scored out of 10), typically requiring only 1–2 sessions to get to their rated comfort level. Meta released a software update which impacted several of the HMDs used in this study part way through the intervention period. This resulted in several login issues or software prompts within the HMD, which were resolved through communication with the research team as required. Six participants had previous experience with a VR HMD for video games; one participant owned their own device. The remaining four participants had no previous experience with VR but ranked their comfort level as 9.5–10 (out of 10) within the first two home sessions.

### System setup

Participants generally found no issues with setting up the VR system at home (Q6 mean = 9.5, M_o_ = 10, scored out of 10), including connecting to the home Wi-Fi and defining a stationary boundary for the virtual environment. Several participants needed to repeat the setup process owing to changing locations during the intervention period (e.g., taking HMD between two households, traveling for vacation), but were able to navigate through the process on their own. Software updates were required for six participants partway through the intervention period owing to a Meta software release, and participants were able to follow the prompts independently to update the HMD or were in contact with the research team by email if they experienced login issues. One participant encountered an error message that tracking was turned off, which prevented the visual rehabilitation application from being launched in the HMD. It was discovered through troubleshooting (by email and phone) with a member of the research team that the participant had tried using the device in a room with the lights off. The issue was resolved by ensuring adequate room lighting was available, so that the outer cameras on the HMD could perform tracking. Finally, the research team followed up with one participant when no data was received from the HMD over Wi-Fi after the first scheduled home-based training session. It was unclear why data from the initial session had not been successfully transmitted but subsequent results were received from the HMD as expected.

### System usability

The system usability scale (SUS) provides a quick and reliable way to evaluate usability.^27^ The scale comprises 10 Likert questions, each with five response options ranging from (1) Strongly Agree to (5) Strongly Disagree. An overall system usability score can be calculated and ranges from 0 to 100, with scores >68 considered above average.

**FIG. 3.**
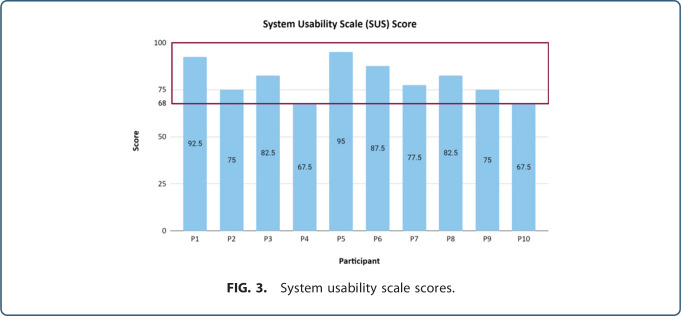
System usability scale scores.

Participants completed the SUS as part of their exit interview (see Appendix A) and scores ranged between 67.5 and 95 (Fig. 3), indicating an average to above-average usability rating for the VR system.

### Device comfort

Scores from participants regarding the physical comfort of the VR equipment ranged from 5 to 10 (Q11 mean = 7.6, M_o_ = 6, 10, scored out of 10). Participants generally reported no issues with the comfort of the hand controllers; however, all hand controllers were configured for right-handed operation, and one left-handed participant performed the intervention by using the right-hand controller in his left hand. Several participants complained that the HMD felt heavy on their head/face over time during a session, or that their eyes felt blurry by the end of a session from fatigue owing to the stimulation. One participant reported that they have screws in their head and the weight of the HMD would cause discomfort/pain owing to interference, whereas another participant said that the HMD would cause soreness with each session until the issue was resolved when a parent recommended removing their ponytail while wearing the device. One participant commented that the foam material on the inside of their own personal VR HMD was more comfortable against the face than for the Meta Quest 2 used for the study. Three of the participants were regularly wearing glasses but did not use them during training; two preferred not to wear them (i.e., did not try together with the device) and one found that the HMD could not fit over their glasses. None of these three participants reported any issues with blurriness in VR without their glasses.

### Acceptance of the protocol

There was wide variability when participants rated how easy it was to integrate the intervention into their daily lives, ranging from 4 to 10 (Q12 mean = 7.7, M_o_ = 6, 8, 9, 10, scored out of 10). Participants reported typically performing the intervention on the couch or desk (beside which the device could be conveniently stored and charged) or in their bedroom (if they preferred a quiet space). Four participants mentioned needing/using external reminders (either from a parent or by setting on a cell phone) to follow the training protocol.

When evaluating the intervention protocol, six participants felt neutral or in agreement with the frequency of training (i.e., every 2 days), one participant thought the training could occur more often (i.e., every day but with shorter sessions), and three participants would have preferred training less often (e.g., once or twice per week). Participants were then asked about the appropriateness of the duration of the intervention (i.e., number of weeks). Five participants were willing to extend training beyond what they had completed, although two of them would do so only if their ophthalmologist/parent believed there were signs of improvement in visual perception up to that point. One participant was unsure about extending and explained that the issue was less about the home-based training and instead about in-clinic visual assessments that were physically tiring. Four participants did not want to extend training (too boring/repetitive) and one of these participants (P7) later withdrew from the study before the 1-month follow-up because they did not want to repeat the in-clinic visual assessments they found to be long and tiring.

### Overall experience/other feedback

Participants reported that they were generally self-motivated to adhere to the intervention protocol (Q14 mean = 2.9, M_o_ = 3, scored out of 4) but commonly said that it became increasingly challenging to stay engaged/interested toward the end of the training schedule. They cited boredom and eye fatigue during training to be the main reasons. Even so, 8/10 participants, including some who were not willing to extend their own intervention period, were likely to recommend the intervention to another person who was also experiencing low vision (Q17, mean = 8.5, M_o_ = 10, scored out of 10). Some participants (*n* = 5) based this recommendation on having perceived improvements in their own visual perception, whereas others (*n* = 3) who did not notice any change thought it was possible the outcome could be different for others. Two remaining participants would *not* recommend this therapy for others; P7 described the training as “*boring*” and “*annoying*” and felt that it was possible an older adult could be interested in the therapy but did not believe anyone of the same age would enjoy it. The parent of the other participant (P8) described that in the beginning, training felt more “*fresh*” but later felt that it would take “*forever*” and that the child required frequent breaks (e.g., hugs, going for a walk, other emotional support) to continue with the training. The parent also suggested that the intervention was “*maybe just not fun enough*,” and that although training became more familiar over the intervention period, her child did not enjoy it more over time.

Several participants believed the intervention should be gamified with different tasks or rewards to provide incentive (P1, P9, P10). Other suggested improvements included having different/more colored objects (i.e., balls) (P8), making the sound of the moving balls “*more pleasant*” (P7), or changing the background of the objects in the virtual environment to be more visually interesting (P4). When participants were asked about their general interest in using VR post-study, many responded that they would want to use it for video games and/or movies while three participants had no interest. Four participants said they would be willing to try VR again for visual therapy but one specified that they would want a different intervention which was “*more interesting*” (P7).

During the exit interview, participants shared what they *perceived* as the impact of the intervention on their visual perception. Five participants reported that they did not notice any improvement themselves (see Table 3); however, one parent commented that her child seemed to be improving in their reading speed during the visual assessment and believed the intervention to be helping since other visual tests were being completed more quickly (based on observation). The remaining five participants indicated that they detected some improvement *[*e.g., *“When I was doing the assessments, I could see around maybe 20% more*” (P3), *“…when I first started I was barely able to see the left edge of the box (in the assessment) but now I'm able to see the left edge of the box way clearer”* (P4)], however half of them mainly noticed improvements while performing the in-clinic assessments rather than in their daily lives [*“…unless I was seeing something with the assessments then I don't really notice it as much”* (P2)]*.* Interestingly, not all participants who self-reported improvement in vision were shown to be responders based on the Esterman binocular field test which was performed pre- and postintervention (see Table 3). Participant P7, whose post-intervention Esterman binocular field test showed an increase of 8 points among 48 points originally unseen in the blind field,^24^ did not perceive any improvement in vision and was the least likely to recommend the therapy to others.

**Table 3.** Summary of Participant Feedback

ID	Intervention period	Self-Reported improvement in vision?^a^	Identified as Responder from Clinical Test^b^	Willing to Extend Training?	Overall experience Q14	Likely to recommend? Q17
P1	4 weeks	No	No	Yes^c^	3/4	8/10
P2	4 weeks	Yes	No	Yes	4/4	10/10
P3	6 weeks	Yes	Yes	Unsure	3/4	10/10
P4	6 weeks	Yes	Yes	Yes	3/4	9.5/10
P5	6 weeks	Yes	Yes	Yes	4/4	10/10
P6	6 weeks	Yes	No	No	3/4	10/10
P7	6 weeks	No	Yes	No	2/4	4/10
P8	6 weeks	No	No	No	1.5/4	5/10
P9	6 weeks	No	No	No	3/4	9/10
P10	6 weeks	No	Yes	Yes^c^	2/4	9/10

a As perceived by participant.

b Based on pre/post visual assessments using Esterman binocular field test.

c Participant only willing to extend if recommended by a doctor or parent.

## Discussion

Through a combination of open-ended questions and qualitative scales in a semi-structured exit interview, we were able to identify certain successes and limitations for this home-based visual rehabilitation program when used with a pediatric population.

### Cybersickness

VR was generally well-tolerated by participants who seldom reported symptoms of cybersickness when completing their VRISE questionnaires,^24^ consistent with other work evaluating the safety of VR with young children.^28^ Research investigating factors that may contribute to cybersickness reported that symptoms such as nausea and disorientation were generally higher after 10 min of continuous exposure to VR.^29^ As Re:Vision consists of three blocks of audio–visual stimulation, with each block limited to 5 min of continuous stimulation, the risk of cybersickness was expected by the research team to be low since the intervention remained below this critical threshold.

One participant described initial feelings of nausea and dizziness from training with the VR HMD that diminished by the third session (one postintervention VRISE score = 16) and no longer persisted for subsequent training sessions. This trend is consistent with findings that individuals can develop increased tolerance for VR and cybersickness with repeated exposure.^30,31^ A second participant indicated that they commonly completed VR training before bed and experienced headaches overnight or woke up with a headache the next morning. However, the participant’s mother indicated that the child had a history of headaches, and it was unclear to her whether or not the morning headaches were associated with Re:Vision. The participant’s VRISE scores did not reflect the presence of any symptoms, such as headaches, which were not present at the time of completing the questionnaire. This study supports existing evidence indicating an overall high tolerance of VR in the pediatric population, characterized by infrequent occurrences of cybersickness. Therefore, health care providers should leverage this safe and portable technology to explore new avenues of delivering rehabilitation programs to younger patients.

### General usability (technical training, system setup, SUS)

Participants rated the quality of the technical training as high overall and reported feeling very comfortable operating the Meta Quest 2 by the end of two sessions. Participants generally found no issues with setting up the VR system at home, including connecting to the home Wi-Fi and defining a stationary boundary for the virtual environment. SUS ratings of the VR system ranged from average to above-average usability, consistent with high user ratings found in literature for the Meta Quest 2 compared with other VR HMDs.^32^

### Engagement and need for gamification

Although all participants were able to follow the intervention protocol for the entire 4–6 weeks, most complained that the training quickly became “*dull*” (P4), “*repetitive*” (P4, P5, P7), or “*boring*” (P4, P7, P8, P9, P10) as the weeks progressed. The addition of the central fixation point after the first 2 weeks increased the difficulty of these tasks and likely exacerbated any negative attitude toward training (e.g., “*At the beginning I wanted to do it every day, then it got boring after fixation point.*” (P7), “*When there was a glitch without fixation point, I thought that was a privilege [not to have it].”* (P3)). Older pediatric participants generally reported higher satisfaction with Re:Vision, complained less about boredom (e.g., “*Maybe personalizing the experience might be more helpful for younger kids but probably not for me, I was okay with the visuals…*” (P1), and may have been better able to appreciate the potential value of the therapy (e.g., “*Mainly [I] just did it because it would help my vision and potentially help the vision of other people… I didn't need any encouragement, didn't give it a second thought…”* (P4). On the other hand, one participant (P7) commented that they did not think anyone of their age would enjoy the program but perhaps would recommend the therapy to an “older person.”

Holmes et al.^33^ discusses the need for more personalization in game design for rehabilitation to better motivate people when it comes to repetitive or monotonous tasks. Serious games, which are interactive games designed to encourage users to practice a skill or learn a valuable lesson beyond only for the purpose of fun, are becoming useful tools in rehabilitation.^34^ They are effective at engaging/motivating patients, increasing the likelihood of adherence to therapy, or improving the accuracy in testing.^35^ Thus, finding a way to gamify Re:Vision may be especially important for therapies directed at pediatric populations who are often already experienced with video games employing incentive strategies and high-quality graphics.

### Perceived effectiveness

Research has shown a clear link between perceived treatment effectiveness and treatment adherence and satisfaction.^36^ Also in this study, participants appeared more willing to extend training or recommend the therapy to others if they had perceived an improvement in their vision over the course of the intervention. For example, one participant shared *“The thing I enjoyed the most was coming here, and seeing the improvements of how much I've gained in my eyesight back.”* (P2)*.* However, effectiveness as determined through clinical assessments may not always be reflected in patients’ perceptions.^36^ Two participants (P2, P6) were not shown to be responders to the Esterman field test yet self-reported improved visual perception and would highly recommend the therapy to others (rating 10/10). Conversely, two other participants (P7, P10) showed improvement on the Esterman field test but did not perceive improvement, rating their overall experience as *Needing Encouragement to Keep Up with Therapy* (2/4), and one participant did not recommend the therapy to others (4/10). Given the small sample size and the reliance on descriptive data, these findings should be interpreted with caution, but at the same time, this highlights that standard assessment tools may not always adequately account for the patient’s full range of symptoms.^37^ Recognizing this gap may lead to the development of new instruments of evaluation that more suitably capture the patient experience.^37^

### Headset accommodation

There was variability in scores when participants assessed the physical comfort of wearing the HMD, with some finding the fit very comfortable while others complained about the HMD weight (e.g., pressure on face or around head). One participant encountered interference between the HMD straps and their ponytail, causing soreness/headaches during training. Although this issue was resolved simply by removing the ponytail before subsequent training sessions, there are other hair types or hairstyles that may not be as easily accommodated by the headset.^38,39^ Mboya’s^39^ thesis experiment involved testing an Oculus Go on 220 participants, and she argued that the headset design was incompatible with the bigger hairstyling (e.g., braids, locs, headwraps) of many African women. Similar interferences may be faced by participants requiring certain auditory aids (e.g., cochlear implants) or those who wear cultural/religious head coverings and wigs.

### Impact of timing of intervention on patient experience

The time of year when participants began their intervention (e.g., summer versus winter) may have affected the degree to which participants found the intervention convenient or burdensome, impacting their overall experience. Four participants began their intervention during the summer holidays and said that training sessions were sometimes missed or forgotten owing to vacation plans but were usually made up by training on consecutive days; one parent commented that it might be easier to follow the protocol during the school year when schedules were more consistent [e.g., “*…because in the summer we’re all over the place…”* (parent of P4)]. However, those participants who performed the intervention while in school also encountered challenges fitting the training sessions into their weekdays, especially if they had activities/appointments after school (e.g., physiotherapy, tutoring, sports), in which case the VR training occurred in the evenings when they were the most tired. In addition, participants training past the summer months had to miss school for in-clinic assessments, adding to the disruption of their regular routine. Travel to the hospital for testing, particularly during the winter months (for P7–P9), may have further influenced perceptions of burden. Although assessments were imposed for safety and research purposes rather than as a requirement for the intervention, the additional burden of completing multiple long and monotonous tests may have contributed negatively to patients’ overall experience.

### Limitations

This study involved a small sample of participants who were all referred to the study by the same clinicians and presents limitations that impact the generalizability of our findings (e.g., to what degree did age and sex influence a participant’s willingness to train with, or use, VR). Furthermore, the training protocol was extended from 4 to 6 weeks after the first two participants; however, this amendment was deliberately introduced in an effort to maximize the potential benefit for participants who otherwise would not have access to any visual therapy. This pilot study utilized the Children’s Visual Function Questionnaire, a vision-specific tool to evaluate QoL, designed to be completed by the parents of children up to 7 years of age. However, researchers did not control for the questionnaire responder and ultimately these data were excluded from analysis.^24^ As participants were all over the age of 7 years, future studies should consider alternative QoL instruments to be completed directly by the participant, such as the National Eye Institute Visual Functioning Questionnaire-25^40^ or World Health Organization Quality of Life.^41^ Qualitative instruments such as these help to better understand the impact of a participant’s perceived effectiveness on their QoL. Finally, we cannot ignore the possibility of response bias in the semi-structured interviews. Participants typically met briefly with the research coordinator at the clinic to register at the front desk and sometimes engaged in light conversation while waiting for the ophthalmologist or technicians to call them for testing. It is possible that familiarity with the research coordinator by the time of the exit interview (conducted by the same research coordinator in most cases) impacted the feedback given by the participant. Growing familiarity between an interviewer and respondent can lead to more honest feedback on the basis of increased trust, but can alternatively result in response bias owing to a social desire to please.^42^ Efforts were made to remind participants that the coordinator was not part of the design of the intervention and honest feedback was encouraged to understand ways in which the therapy could be improved upon.

## Conclusion

Results from this interview study showed that participants encountered few technical difficulties using Re:Vision and gave average to above-average ratings of usability through SUS. This supports the potential for VR to be used in tele-rehabilitation, providing more equitable and accessible care that would typically only be available through clinics with specialized equipment. All participants were able to complete their training protocol, demonstrating that a 6-week home-based intervention with training every other day is acceptable for a pediatric population and allows for flexibility in accommodating schedules of participants and their families. However, many participants lost enthusiasm over time and general consensus that the therapy was too repetitive suggests that certain design changes, such as incorporating personalized content (e.g., backgrounds, icons) or gamification (e.g., implement a reward system for completing tasks correctly), could better motivate users, especially amongst the pediatric population. Finally, for future studies, it is also essential to understand which factors can contribute to a patient’s perceptions of effectiveness and ensure that meaningful outcomes are captured to accurately reflect the impact on patients’ everyday lives.

## Abbreviations Used

3D-MOT: 3D Multiple-Object Tracking
BCVA: Best Corrected Visual Acuity
BTH: Bitemporal Hemianopia
CVFQ: Children’s Visual Function Questionnaire
HH: Homonymous Hemianopia
HMD: Head Mounted Display
QoL: Quality of Life
REB: Research Ethics Board
SUS: System Usability Scale
UHN: University Health Network
VFQ-25: Visual Functioning Questionnaire - 25
VRISE: Virtual Reality Induced Symptoms and Effects
WHOQOL-BREF: World Health Organization Quality of Life Brief Version
